# Preliminary experience and learning curve of endoscopic nipple-areolar-complex sparing total mastectomy: A single-center retrospective study

**DOI:** 10.1371/journal.pone.0311764

**Published:** 2025-01-09

**Authors:** Da young Yu, Tae Yul Lee, Duk Woo Kim, Young Woo Chang, Gil Soo Son, Hye Yoon Lee

**Affiliations:** 1 Division of Breast and Endocrine Surgery, Department of Surgery, Korea University College of Medicine, Seoul, Korea; 2 Department of Plastic Surgery, Korea University College of Medicine, Seoul, Korea; Gangnam Severance Hospital, Yonsei University College of Medicine, REPUBLIC OF KOREA

## Abstract

**Background:**

In this study, the preliminary experience of endoscopic nipple-areolar-complex (NAC) sparing total mastectomy were analyzed and reported.

**Methods:**

The medical records of the patients who underwent Endoscopic NAC sparing total mastectomy from November 2019 to June 2022 in a single institute were collected and analyzed. The medical records of their clinicopathologic characteristics, perioperative parameters, postoperative complications, oncologic results were collected retrospectively. The learning curve was evaluated using univariable and multivariable analyses and analyzed using the CUSUM method.

**Results:**

A total of 56 surgeries from 53 patients were analyzed. The mean total operation time was 272.50±66.74 minutes while mean oncologic mastectomy was 155.61±43.21 minutes. The mean postoperative hospital days were 9.85±4.70 days. In CUSUM analysis, the cases needed to decrease operation time were 10th. The overall complication rate related to implant was 15.09%. There were one locoregional recurrence patient and one distant metastasis and mortality patient observed during median follow-up of 19.12±12.01 months.

**Conclusion:**

According to our preliminary study, endoscopic NAC sparing total mastectomy is a safe procedure, and the operation time can be reduced with accumulated surgical experience.

## Introduction

Traditionally, breast surgery was commonly performed as an open procedure without any doubt [1]. The breasts, being protruding dome-shaped organs, are easily accessible on the surface of the body. However, over time, cosmetic concerns have gained increasing importance, even in oncologic surgery [2, 3]. In response to these demands, numerous innovative novel surgical technologies have been invented, including the endoscopic approach.

In the beginning stage of endoscopic mastectomy, some surgeons employed only an endoscopic camera to enhance visualization during surgery, while most other procedures remained almost the same as traditional open surgery [4]. Endoscopic surgical instruments were introduced more recently, in addition to the visualization provided by the endoscopic camera [5].

Following this trend, our institution also initiated the practice of endoscopic nipple-areolar-complex(NAC) sparing total mastectomy with concurrent reconstruction. As with any emerging technology, the learning curve is a crucial consideration.

In this study, we report the preliminary experience of endoscopic nipple sparing total mastectomy performed by the two surgeons at a single institute. We also analyzed the learning curve of a single surgeon using the cumulative sum (CUSUM) method [6]. Patients’ characteristics and potential factors affecting operation time were also evaluated.

## Materials and methods

### Patients

The study was conducted retrospectively and included patients who had undergone endoscopic nipple-areolar-complex (NAC) sparing total mastectomy between November 2019 and June 2022 at Korea University Ansan Hospital, a tertiary medical center in Korea. Total mastectomy was performed for patients with multifocal or multicentric breast cancer. For those with a single lesion, candidates for the mastectomy included those with a cancer diameter over 30mm or those with coexisting diffuse calcification. A total of 56 surgeries from 53 patients were reviewed, including 3 patients who had undergone bilateral breast surgery.

Data were collected for each patient, including their clinicopathologic characteristics based on the pathologic report and medical records, method of breast reconstruction, operative time, length of hospital stay, complications, accumulated amount of drainage, extracted specimen weight, volume of inserted implant, and median duration of follow-up. Complications occurring within 4 weeks after surgery were defined as early complications, while complications occurring after 4 weeks were defined as late complications. Complications included all events that occurred at the surgical site postoperatively and encompassed inflammation, necrosis, contracture, and implant-related complications. All patient data were collected from August 1, 2023, to September 30, 2023.

This retrospective study was approved by the Institutional Review Board of Korea University Ansan Hospital (approval number: 2023AS0062), and a waiver of informed consent was requested and approved.

All patients included in the study underwent endoscopic NAC sparing total mastectomy performed by two surgeons, either LHY or CYW. Concurrent reconstruction surgery was performed after the mastectomy. The study included patients with both ductal carcinoma in situ (DCIS) and invasive ductal carcinoma (IDC).

When analyzing the learning curve, however, we focused on 29 surgeries performed by a single surgeon named LHY. Although a total of 44 out of 56 surgeries were initially considered, we excluded 15 cases for the following reasons. A total of 6 cases with 3 patients who received bilateral mastectomy were excluded due to the lack of recorded operation time for each side. Additionally, 9 patients who required further axillary lymph node dissection were also excluded as there was no available information about time on the completion of endoscopic mastectomy and the initiation of axillary dissection. This analysis intentionally concentrated only on the procedures performed by LHY to ensure consistency and accuracy in assessing the learning curve.

### Surgical procedure

All patients were operated on in the same sequence, and detailed description of the surgical procedure is mentioned in the previous study [7]. Patients were placed in a supine position with both arms abducted on the arm boards. The contralateral arm was abducted for reconstruction surgery because bilateral breasts should be compared after the insertion of the implant.

An approximately 5cm single incision was made at the lower, anterior axillary line, starting from the inferior mammary fold. The sentinel lymph nodes were identified first using a technetium-99m radioisotope detector and excised through this incision and sent to the pathology department for frozen biopsy. The incision site was then prepared for the insertion of a Glove port (Nelis Corporation, Bucheon, Korea) to protect the skin from injury during surgery and provide multiple ports for the endoscopic surgery through a single incision. A 3cm radius of skin flap around the incision on both the upper and lower borders of the breast was elevated. Before the insertion of Glove port, approximately 250mL of tumescent solution was infiltrated into the subcutaneous fat layer of the breast with a Veress needle through this workspace.

After creating a port site and before starting an endoscopic mastectomy, a sentinel lymph node biopsy was performed on all patients. All sentinel lymph node biopsies were conducted using the open method through a 5 cm incision made for port insertion. For all sentinel lymph node biopsies, a frozen section was used to check for lymph node metastasis. If lymph node metastasis was confirmed, an axillary lymph node dissection was performed using the open method through the port insertion incision after completing the endoscopic mastectomy.

Initially, blunt dissection using a straight tunneler was performed for the first few surgeries. However, we soon stopped blunt dissection to maximize the advantages of endoscopic surgery. Instead, we performed meticulous dissection using endo scissors later.

After inserting the Glove port, a 90-degree 10mm flexible endoscope (ENDOEYE FLEX 10mm, LTF-S190-10; Olympus Corporation, Tokyo, Japan) was inserted through the largest port, while an endoscopic dissector and an electrocautery were inserted through other separate ports. With guidance from the endoscopic camera, lifting the lateral breast at the anterior border of the latissimus dorsi muscle was done to approach the retromammary space. With the assistance of carbon dioxide (CO_2_) gas pressure maintained at approximately 6 mmHg during the surgery, the breast tissue was easily lifted with one hand, while the other hand performed further dissection medially to the edge of the sternum, superiorly to the level of the clavicle, and inferiorly to the thoracoabdominal aponeurosis.

After dissecting the retromammary fat layer, the Glove port was repositioned, with the breast tissue placed at the bottom. Dissection between breast tissue and the subcutaneous fat layer along Cooper’s ligament was performed. The use of an endoscopic camera provided visual assistance, allowing for meticulous and precise dissection. This enabled clearer dissection of the layer, as well as effective coagulation and cutting of small blood vessels and subareolar mammary ducts. When encountering the subareolar mammary duct, the nipple margin was dissected separately and sent to the pathology department for frozen section analysis.

After the dissection progressed along the dome shaped breast tissue, the mastectomy was considered complete when the dissection met the previously dissected retromammary space. The breast tissue was then extracted through the single incision and marked with sutures to indicate the direction.

### Statistical analyses

We performed a two-sample t-test or Mann-Whitney test to compare continuous variables between two groups, and reported the statistics as mean and standard deviation. Statistical significance was defined as a p-value less than 0.05, and all tests were two-tailed. Linear models were applied to examine the differences between two groups after adjusting for covariates such as specimen and BMI. All statistical analyses were conducted using R (version 4.2.3; R Foundation for Statistical Computing, Vienna, Austria).

CUSUM is a statistical method used to explore trends in data. It is a method for detecting when changes in data have exceeded or fallen below a certain threshold. CUSUM is particularly useful for examining changes in data over time. To do this, we subtracted the mean value of each time point’s data and calculated the cumulative sum to track changes in the data.

## Result

### Clinicopathological characteristics

During the study period, a total of 53 female patients underwent 56 surgeries conducted by two surgeons. The mean age at the time of operation was 48.96±8.00 years, and the mean BMI was 24.15±3.44kg/m^2^. Three out of the total 53 patients underwent bilateral endoscopic nipple-areolar complex sparing mastectomy (NSM). The side of the breast cancer was evenly distributed on each side, accounting for 50% on either side. Of the patients, 21 (37.5%) were diagnosed with ductal carcinoma in situ (DCIS), while the others were diagnosed with invasive ductal carcinoma (IDC).

Among the patients, 44 (82.7%) underwent sentinel lymph node biopsy only, while 9 (13.0%) underwent additional axillary lymph node dissection.

Two of the patients received neoadjuvant chemotherapy before surgery, and no patient received hormonal therapy, anti-Her2 targeted therapy or radiotherapy, preoperatively.

Patients diagnosed with pN1 or pN1mi who did not undergo axillary lymph node (LN) dissection were those who tested negative on sentinel lymph node (SLN) frozen biopsy, but were later found to be positive on final permanent pathology. Further clinicopathologic characteristics of patients received endoscopic NAC sparing mastectomy for breast cancer were summarized in Table 1.

**Table 1:** Patients’ clinicopathological characteristics.

	n	range
Median F/up (months, median±SD)	19.12	2.37–45.63
Gender (Female)	53 (100%)	
Age (years, mean±SD)	48.96±8.00	30–66
Height (cm, mean±SD)	158.18±5.14	142.5–168.9
Weight (kg, mean±SD)	60.43±8.95	43.16–85.80
BMI (kg/m^2^, mean±SD	24.15±3.44	18.07–34.68
Location (Right/Left)		
Left	28 (50%)	
Right	28 (50%)	
Bilateral	3	
Multiplicity		
Multifocal	11 (19.6%)	
Multicentric	23 (41.4%)	
Single lesion	22 (39.3%)	
Clinical T stage		
cT0	27 (48.2%)	
cT1	14 (25%)	
cT2	14 (25%)	
cT3	1 (1.8%)	
Clinical N stage		
cN0	45 (80.4)	
cN1	11 (19.6%)	
Pathologic T stage		
pTis	21 (37.5%)	
pT1	20 (35.7%)	
pT2	13 (23.2%)	
pT3	2 (3.6%)	
Pathologic N stage		
pN0	44 (78.6%)	
pN1mi	1 (1.8%)	
pN1	6 (10.7%	
pN2	5 (8.9%)	
Pathologic Stage		
0	20 (35.7%)	
IA	15 (26.8%)	
IB	1 (1.8%)	
IIA	11 (19.6%)	
IIB	3 (5.45%)	
IIIA	6 (10.7%)	
Mastectomy type		
Endoscopy assisted NSM	54 (96.4%)	
Endoscopy assisted SSM	2 (3.6%)	
Axillary surgery		
SLNB only	44 (82.7%)	
SLNB then ALND	9 (13.0%)	
Not done	3 (4.3%)	
Grade		
I	12 (21.4%)	
II	29 (51.8%)	
III	14 (25.0%)	
Unknown	1 (1.8%)	
ER		
Negative	16 (28.6%)	
Positive	40 (71.4%)	
PR		
Negative	18 (32.1%)	
Positive	38 (67.9%)	
HER2		
Negative	39 (69.6%)	
Overexpressed	16 (28.6%)	
N/A	1 (1.8%)	
Neoadjuvant Chemotherapy	2 (3.6%)	
Adjuvant Chemotherapy	20 (35.7%)	
Adjuvant Hormonal Therapy	38 (67.9%)	
Adjuvant Anti-HER2 targeted therapy	5 (8.9%)	
Adjuvant Radiotherapy	6 (10.7%)	

Abbreviations: SD, standard deviation; BMI, body mass index; NSM, nipple-sparing mastectomy; SSM, skin-sparing mastectomy; SLNB, sentinel lymph node biopsy; ALND, axillary lymph node dissection; ER, estrogen receptor; PR, progesterone receptor; HER2, human epidermal growth factor receptor 2; N/A not applicable

### Perioperative details

Reconstruction surgeries were performed by plastic surgeons. Among the patients, 53 (94.6%) underwent direct-to-implant reconstruction, while 1 patient (1.8%) underwent tissue expander insertion, and 2 patients (3.6%) underwent transverse rectus abdominus myocutaneous (TRAM) flap reconstruction. The median operation time for mastectomy was 140 minutes, range from 90 minutes to 280 minutes. The mean specimen weight was 331.27±140.56g (87–656). The median postoperative hospital days were 11 days, range from 3 days to 28 days. (Table 2).

**Table 2:** Perioperative parameters.

	Number	%
Reconstruction method		
Direct-to-implant	53	94.6%
Tissue expander	1	1.8%
TRAM reconstruction	2	3.6%
	Mean±SD	Median (range)
Operation time (min)		
Total	272.50±66.74	163.5 (161–525)
Mastectomy	155.61±43.21	140 (90–280)
Reconstruction	116.89±53.83	108.5 (42–330)
Mean mastectomy weight (g)	331.27±140.56	
Reconstruction implant volume (ml)	318.91±85.68	
Postoperative hospital days (days)		11 (3–28)

Abbreviations: TRAM, Transverse rectus abdominis muscle; SD, standard deviation

The overall complication rate was 10 out of 56 cases (17.9%), with 8 of the 10 cases involving implants. The complications were classified based on their symptoms and the time of onset after surgery. Overall complications were classified according to the treatment received. Early and late complications were distinguished based on whether they occurred within 30 days postoperatively and were further classified by symptoms. Among these cases, four patients (7.6%) required surgical intervention, including implant removal or change, with two of them occurring within the first 30 days after surgery. The remaining cases required only local debridement or antibiotics. (Table 3).

**Table 3:** Perioperative complications.

	n (total = 56, implant = 53)	Treatment
Overall	10/56 (17.9%)	
Implant loss	4/53 (7.6%)	
Implant change	3/53 (5.7%)	
Implant removal	1/53 (1.9%)	
Early	5/56 (8.9%)	
Partial ischemia of the NAC	1	Debridement and IV antibiotics
Infection	2	IV and PO antibiotics
Implant change	2	
Late	5/56 (8.93%)	
Contracture	2	No further treatment
Implant rupture	1	Implant change
Skin flap necrosis	1	Debridement and IV antibiotics
Infection	1	Implant removal

Abbreviations: NAC, nipple areolar complex; IV, intravenous; PO, per os

### Outcome and morbidity details

Of all the surgeries performed, it was discovered that two patients had positive nipple margins, despite being informed that their nipple margins were negative based on frozen section analysis during the surgery. Both of them underwent additional NAC excision at a later time. On permanent pathology, one patient was found to have a positive inferior margin, despite the frozen biopsy being negative. Since there was no remaining breast tissue that could be resected, the patient received additional radiotherapy.

During the 19.12±12.01 months of follow-up period, only one patient experienced locoregional recurrence. This patient was one of the patients whose permanent sentinel LN biopsy was revealed as positive. Ipsilateral axillary LN enlargement was diagnosed during a routine checkup, and the patient subsequently underwent axillary LN dissection.

There was one mortality during the follow-up period. This patient received adjuvant chemotherapy but refused to undergo anti-Her2 targeted therapy due to economic constraints. Distant metastasis to the lungs, bones, brain, and liver were diagnosed in the 13.7^th^ month postoperatively, and the patient passed away in the 20.8^th^ month despite receiving palliative radiotherapy, chemotherapy, and anti-Her2 targeted therapy. (Table 4).

**Table 4:** Oncologic results.

	n (total = 53)	Description
Margin involvement (n, %)	4 (7.6%)	
Positive nipple margin in frozen	1 (1.9%)	NAC sacrifice during operation
Positive nipple margin in permanent	2 (3.8%)	2^nd^ operation for NAC scarification
Positive breast margin in permanent	1 (1.9%)	Adjuvant radiotherapy
Locoregional recurrence (n, %)	1 (1.9%)	
Distant metastasis (n, %)	1 (1.9%)	
Mortality (n, %)	1 (1.9%)	

Abbreviations: NAC, nipple areolar complex

### Learning curve

To evaluate the surgeon’s efficiency and learning curve, the patients who underwent unilateral NSM without further axillary lymph node dissection by single surgeon named LHY were extracted. Total 29 patients were analyzed and divided into two groups: Group 1 consisting of the first ten patients and Group 2 consisting of the 19 most recent patients. The mean operation time for Group 1 was 169.60 ± 28.45 minutes, while that for Group 2 was 144.89 ± 28.25 minutes. A univariate analysis revealed a significant time difference between the two groups, with a p-value of 0.034031. As BMI and extracted specimen weight were considered factors that could significantly affect the difficulty and total duration of surgery [8], they were defined as confounding factors and a multivariate analysis was conducted. As a result, we could find out that the p-value was 0.007380 (Table 3).

**Fig 1:**
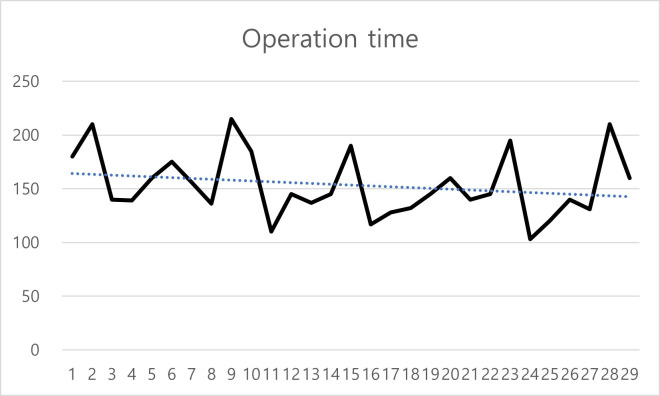
Operation time by chronological case sequence. Operation time, defined as the duration from the first incision to the extraction of the resected specimen, is presented in minutes according to the chronological sequence of cases.

**Fig 2:**
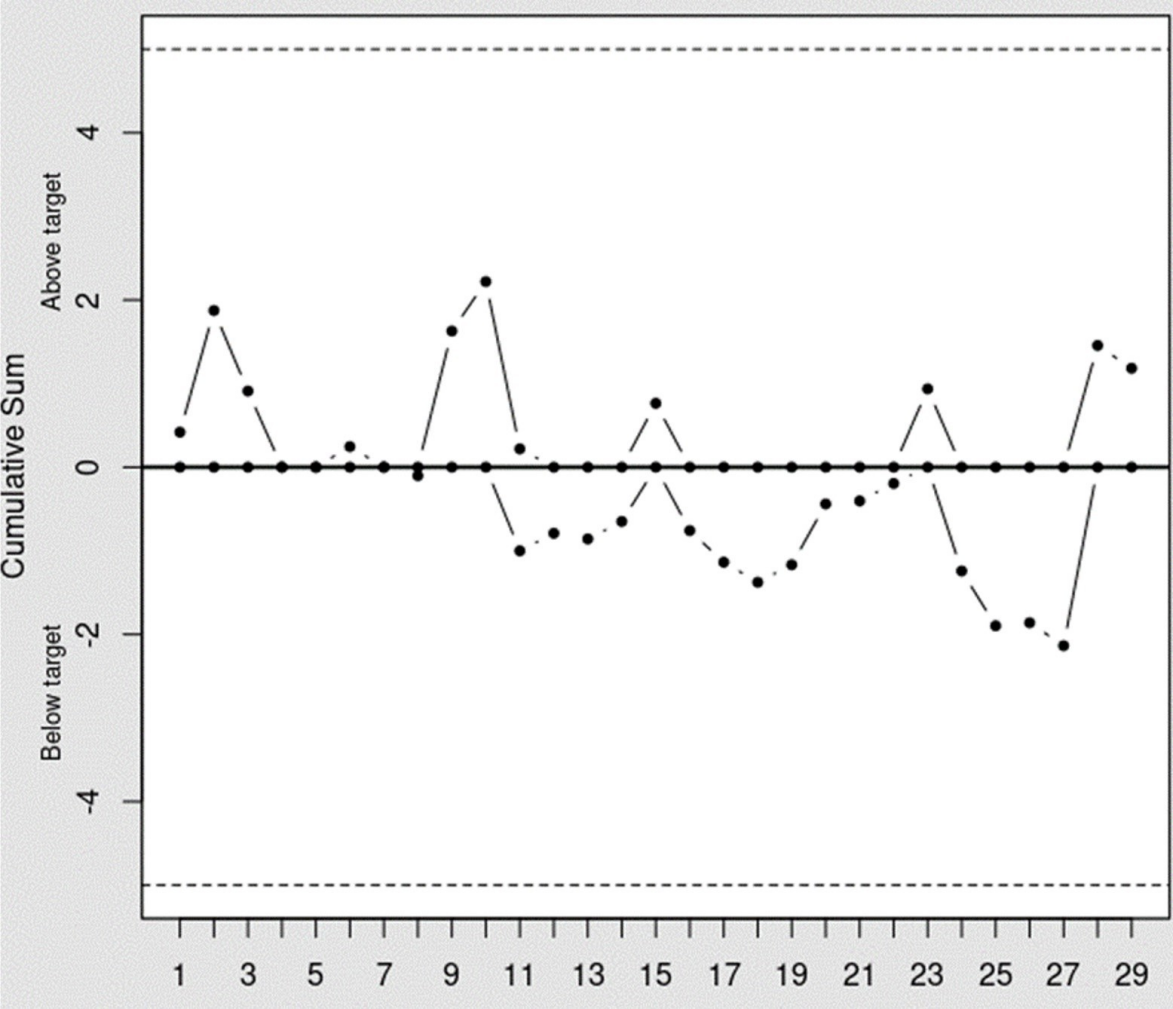
CUSUM chart analysis of operation time. CUSUM chart analysis reveals that the accumulated mean operation time falls below the overall mean operation time starting from the tenth surgery.

This learning curve could also be visualized through X bar and CUSUM chart (Figs 1 and 2). As shown in Fig 1, the operation time exhibited a tendency to decrease with an increasing number of experience. Additionally, while the CUSUM chart analysis indicated that the accumulated mean operation time tended to remain below the total mean operation time after the 10th surgery (Fig 2).

## Discussion

This study presents an analysis of data collected consecutively on the experience of performing endoscopic mastectomy at a single institution. The primary focus of the analysis was the learning curve related to the surgical time of a single surgeon. The findings revealed a statistically significant reduction in surgical time after surpassing 10 cases. Specifically, the difference in surgical time between the initial 10 cases and the subsequent 19 cases was approximately 25 minutes, demonstrating a significant improvement. This finding aligns with the levels reported in other previously published studies. According to research by Lai et al. from Taiwan, a decrease in surgical time was observed following the first 15 cases [6, 8, 9]. Consequently, it can be suggested that those beginning endoscopic mastectomy may experience a reduction in surgical time after approximately 10 to 15 cases.

Our previous study confirmed that endoscopic mastectomy requires a longer operative time compared to conventional open mastectomy [7]. However, it was also found through analysis that this increase in operative time can decrease after 10 to 15 cases, and that the prolonged operative time does not significantly affect the occurrence of complications.

The study also evaluated the safety of endoscopic mastectomy. Both early and late postoperative complications were assessed, revealing an overall complication rate of 17.9%. This was comparable to the complication rates reported in previous reported other study [8, 10]. Despite the relatively short follow-up period, oncologic safety was confirmed. Although there was one case of recurrence and death, this patient was at stage IIIA at the time of diagnosis, suggesting that the issues were not related to the surgical procedure itself. Excluding this patient, the oncologic safety results were comparable to those reported in other studies [8, 10].

The technique of endoscopic mastectomy introduced in the 1990s [11] and has since evolved into robotic mastectomy. However, in countries such as Korea and other parts of Asia, robotic mastectomy is more commonly performed, and there is a greater volume of related literature [12–15]. This preference may be due to the higher labor demands associated with endoscopic surgery compared to robotic surgery. This study aims to emphasize the advantages of endoscopic mastectomy.

When comparing endoscopic mastectomy with robotic mastectomy, endoscopic mastectomy offers several advantages. Firstly, it is more economical [6]. Although additional analysis and future publications are anticipated, endoscopic surgery offers significant cost advantages over robotic surgery, with pricing like open surgery. Many breast cancer patients require various treatments post-surgery, often including expensive chemotherapy agents [16]. Choosing a more cost-effective surgical approach can be significant in managing overall medical expenses.

Secondly, endoscopic surgeries are time-efficient [13]. Unlike robotic mastectomy, they do not require docking, and the process of changing surgical tools is quicker and simpler.

Lastly, during endoscopic surgery, the surgeon’s tactile sensation can guide the control of skin flap thickness while dissecting the medial portion of the breast. The dome-shaped nature of the overlying skin makes it susceptible to damage or perforation without careful consideration. Endoscopic mastectomy allows for direct visualization and palpation of the operative field, facilitating safe assessment of flap thickness and surgical progress—capabilities that robotic surgery does not offer.

Conversely, endoscopic mastectomy has certain limitations compared to robotic surgery. The instruments used in endoscopic procedures lack joints, resulting in less ergonomic handling and frequent collisions between instruments, a common issue with single-port endoscopic surgery. Nonetheless, as indicated by the learning time analysis results in this study, improved proficiency with endoscopic techniques and equipment is expected to reduce these inconveniences and, consequently, the surgical time. Considering both the advantages and limitations of endoscopic mastectomy, it remains a viable option for breast cancer surgery.

However, it’s important to acknowledge the limitations of our study. Our research was conducted with a retrospective design within a single center. The study included a small number of patients, and it was a one-arm study. Further randomized prospective studies with larger sample sizes and involving more surgeons are needed to confirm the efficiency and safety of this technology.

## Conclusion

As with any novel technology, the initial introduction of endoscopic surgery into breast surgery presented challenges. However, it has been demonstrated that this new technology is safe, and with repetition and practice, the surgeon can become more skilled and the operation time can be reduced.
